# The Clinical Features and Risk Factors of Parenchymal Neuro-Behcet's Disease

**DOI:** 10.1155/2019/7371458

**Published:** 2019-09-12

**Authors:** Dong Yan, JinJing Liu, Yuehua Zhang, Wei Yuan, Yan Xu, Jing Shi, Chaoran Li, Yining Wang, Linyi Peng, Yunjiao Yang, Jiaxin Zhou, Di Wu, Zhichun Liu, Xiaofeng Zeng, Fengchun Zhang, Wenjie Zheng, Yan Zhao

**Affiliations:** ^1^Department of Rheumatology and Clinical Immunology, Peking Union Medical College Hospital, Clinical Immunology Center, Chinese Academy of Medical Sciences and Peking Union Medical College, the Ministry of Education Key Laboratory, Beijing 100730, China; ^2^Department of Rheumatology and Immunology, The Second Affiliated Hospital of Soochow University, Suzhou 215004, China; ^3^Department of Oncology and Immunology, The Fourth Hospital of Hebei Medical University, Shijiazhuang 050011, China; ^4^Department of Rheumatology, Kailuan General Hospital, Hebei United University, Tangshan 063000, China; ^5^Department of Neurology, Peking Union Medical College Hospital, Beijing 100730, China; ^6^Department of Radiology, Peking Union Medical College Hospital, Beijing 100730, China

## Abstract

To investigate the clinical features of parenchymal neuro-Behcet's disease (p-NBD), we retrospectively reviewed the medical records of 1009 BD patients admitted to Peking Union Medical College Hospital from 2000 to 2016. Forty-two patients (25 males and 17 females) with p-NBD and eighty-four age- and sex-matched BD patients without neurological involvement who were served as controls were enrolled. Neurological onset was concomitant with the onset of BD in six cases (14.3%). Pyramidal signs (50.0%) and headache (33.3%) were the most common manifestations. On MRI, the lesions were mainly in the midline structures and hyperintense in the T2-weighted image. The most common lesion was the brainstem (54.8%). Spinal cord involvement was observed in five cases, four of which with cervical cord involvement. Multifocal lesions were observed in 13 patients. Ocular involvement was more prevalent in p-NBD (35.7%) (*P* = 0.041, OR = 2.36, 95% CI = 1.03-5.44) compared with controls. All patients received corticosteroids and immunosuppressants, mainly cyclophosphamide (39/42). Six patients with severe/refractory condition received biological agents and achieved response measured by decreased Rankin score (*P* = 0.002). With a median follow-up of 28 months, 22 patients (61.1%) achieved clinical improvements, while 10 (27.8%) relapsed and 4 died (mortality rate 11.1%). p-NBD is a rare yet disabling and life-threatening complication of BD. Ocular involvement is a risk factor for p-NBD. Promptly aggressive treatment is essential for improving prognosis, and biological agents might be a promising approach for severe/refractory p-NBD.

## 1. Introduction

Behcet's disease (BD) is a multisystem inflammatory disease with unknown etiology. Central nervous system involvement in BD, the so-called neuro-Behcet's disease (NBD), is one of its most serious complications and an important cause of morbidity and mortality [1]. The frequency of NBD greatly varies from 1.3% [2] to 59% [3], due to differences in ethnic, geographical distribution, and study designs. It can be caused by either primary neural parenchymal lesions or secondary to vascular involvement. The former is called parenchymal NBD (p-NBD) and represents the majority of NBD [4–6]. According to the site of the lesions, p-NBD can be classified as multifocal/diffuse, brainstem, spinal cord, cerebral, asymptomatic, and optic neuropathy [7]. It may present with numerous manifestations, such as pyramidal signs, headache, and dysarthria, consistent with the site of the lesions. The 10-year mortality of p-NBD is 10% [7], while nonparenchymal NBD (non-p-NBD) often tends to recover well with appropriate and prompt treatment.

To date, p-NBD has been reported in some other countries [4, 8–12] and the clinical features of Chinese p-NBD patients have not been characterized by clarity. To address this issue, we conducted a retrospective study on hospitalized BD patients and identified the patients with p-NBD. We summarized the clinical characteristics, the cerebrospinal fluid (CSF) tests, magnetic resonance imaging (MRI) findings, treatment, and prognosis and further explored the potential risk factors for p-NBD in order to make an early diagnosis and improve prognosis.

## 2. Materials and Methods

### 2.1. Patients

BD patients who were admitted to Peking Union Medical College Hospital from 2000 to 2016 were retrospectively enrolled. All patients fulfilled 1990 International Study Group (ISG) BD criteria [13] or new International Criteria for BD (ICBD) [14]. The diagnosis of neurological involvement was based on the abnormalities on neurological examination, CSF analysis, or neuroradiological examinations. The diagnosis was made by two rheumatology experts and two neurology experts according to the criteria of the 2014 International Consensus on NBD [15]. We chose the modified Rankin score to assess the disability status of NBD patients [16]. Clinical data including demographics, clinical features, laboratory tests, imaging, treatment, and outcome were retrospectively extracted from the medical records.

The courses of p-NBD were classified as the acute course (defined as acute onset of neurological symptoms and signs lasting >24 hours), chronic progressive course, and silent course (defined as detection of abnormal findings on neurological examination in cases who did not have any neurological complaints) [11].

We randomly matched eighty-four BD patients (at 1 : 2 ratio) without neurological involvement by sex and age as the control group to identify the risk factors in p-NBD. The study was reviewed and approved by the institutional ethics review board of Peking Union Medical College Hospital in accordance with the Declaration of Helsinki. Given the study was based on the review of medical records, written informed consent was waived. The patient's records/information was anonymized and deidentified prior to the analysis.

### 2.2. Statistical Analysis

Statistical analysis was performed with SPSS version 21.0 (IBM Inc., Armonk, USA). Frequencies and percentages were used for categorical variables. Mean ± standard deviation (SD) was used to express quantitative variables of the normal distribution, while median and range were used for abnormal distribution. The continuous variables were analyzed by *t*-test or Mann–Whitney *U* test, as appropriate. A value of *P* < 0.05 was regarded as indicating statistical significance.

## 3. Result

### 3.1. Epidemiology

Of 1009 hospitalized BD patients in the same academic hospital during the same period, NBD was documented in 62 patients (6.1%), and a total of 42 patients had parenchymal involvement (4.2%, 25 males and 17 females). In patients with p-NBD, the male/female ratio was 1.47 : 1. Their age at onset of BD was 30.0 ± 11.1 years old, and age at neurological onset was 35.3 ± 12.1 years old. Most of the patients suffered neurological symptoms after other initial systemic symptoms of BD in the median interval of 2 months (range from 0 to 18 months). Neurological onset was concomitant with the onset of BD in six cases (14.3%). Additionally, of the 20 NBD patients but without p-NBD in our cohort, 17 patients were presented with cerebral venous sinus thrombosis (CVST) and the other three patients had peripheral nervous system involvement.

### 3.2. BD Manifestations

In patients with p-NBD, oral ulceration presented in all, followed by genital ulcers (64.3%), skin lesions (45.2%) (including pseudofolliculitis, erythema nodosa, or positive pathergy test), ocular involvement (35.7%) (including uveitis, retinal vasculitis, and scleritis), arthritis (21.4%), vascular involvement (14.3%) (deep vein thrombosis in 5 cases and pulmonary embolism in 1 case), and gastrointestinal involvement (7.1%) (all presented as terminal ileum ulceration). Only one case had mitral valve lesion (Table 1).

## 4. Neurological Features

### 4.1. Clinical Features

Seventeen patients (40.5%) presented with an acute onset and 24 patients (57.1%) had a progressive course, while one case showed a silent course. The most common involved sites of p-NBD were brainstem (23/42, 54.8%) and hemisphere (22/42, 52.4%). Five cases (11.9%) had spinal cord involvement, in which 4 had cervical cord involved and one had thoracic cord involved. 13 cases (31.0%) suffered from multiple lesions (Table 2). The diagnosis of p-NBD in 3 cases was based on the typical neurological symptoms and signs, abnormal CSF examinations, and electroencephalogram, though neuroradiological findings were normal. In addition to BD, no other diagnosis could better explain the clinical picture.

Patients with p-NBD had a variety of neurological signs and symptoms (Table 3). The most common clinical symptoms were pyramidal signs (50.0%), followed by headache and psychological and behavioral change (each 33.3%). Irritating cough (31%), myasthenia (28.6%), dysarthria (26.2%), and movement disorder (21.4%) were also commonly observed. Some symptoms were less frequently noticed such as urinary retention, visual loss, cognitive dysfunction, epilepsy, conscious disturbance, ataxia, and hemiplegia.

### 4.2. Neurological Imaging

All patients received neurological MRI examination. Thirty-nine cases presented with parenchymal lesions. Of those with parenchymal impairment, the brainstem, the periventricular area, the centrum semiovale, and the spinal cord were involved in twenty-two (22/42), thirteen (13/42), eight (8/42), and five patients (5/42), respectively. Typically, the lesions were hyperintense on T2-weighted MRI and hypointense on T1-weighted sequences. Only one patient had brainstem atrophy, whose interleukin-6 (IL-6) level in CSF was elevated (78.5 pg/mL).

### 4.3. CSF Analysis

CSF analysis was performed in 40 patients. The opening pressure was increased in 8 cases (20%), ranging from 195 to 270 mmH_2_O. CSF protein was mildly elevated in 22 cases, with a mean level of 0.51 ± 0.24 g/L. All cases showed normal chloride and glucose level of CSF. Pleocytosis was shown in 11 cases, and assessment of cytological examination of CSF was conducted in 20 patients. Nine cases showed mixed pictures with lymphocyte predominance, and two cases showed mainly neutrophil picture. Both CSF and serum protein electrophoresis for oligoclonal bands (OCB) were performed in 21 cases. Seven cases were positive in CSF while negative in serum (33.3%). CSF cytokine levels were tested in three cases during acute attacks. Elevated IL-6 and tumor necrosis factor (TNF-*α*) levels were detected in two patients, respectively.

### 4.4. Laboratory Examinations

Erythrocyte sedimentation rate (ESR) was significantly elevated in 45.2% (19/42) with the median level of 15 (ranged 0-85 mm/h). While hypersensitive C-reactive protein (hs-CRP) levels elevated in 50% (21/42) with the median level of 3.0 (0.14-124 mg/L). All patients but one showed low-titer antinuclear antibody (ANA) (1 : 160).

### 4.5. Comparison between Acute and Progressive p-NBD

The data showed no statistical significance in clinical features, sites of neurological involvement, CSF tests, and laboratory examinations between acute and progressive p-NBD patients (*P* > 0.05) (data not shown).

### 4.6. Comparison with BD without Neurological Involvement

Eighty-four age- and gender-matched BD patients without neurological involvement were randomly selected at 1 : 2 ratio and served as the control group. Compared with the controls, the prevalence of ocular involvement (including uveitis, retinal vasculitis, and scleritis) was significantly higher in p-NBD (35.7%) (*P* = 0.041, OR = 2.36, 95% CI = 1.03-5.44) (Table 4). The average interval from the onset of ocular to neurological symptoms was 42.7 ± 38.1 months. Fifteen cases had ocular involvement, among whom six cases were treated with cyclosporine A (CsA) before the onset of neurological symptoms with the dosage ranging from 75 mg bid to 150 mg bid for (36.2 ± 36.1) months. Some other clinical manifestations, ESR, and CRP showed no statistical significance.

### 4.7. Treatment and Outcome

All p-NBD patients received corticosteroids (as prednisone ≥ 1 mg/kg/d or the equivalent dosage of other corticosteroids) and 23 patients (54.8%) received methylprednisolone pulse therapy. A total of forty patients were treated with immunosuppressants, in which cyclophosphamide (CTX) 39 cases, CsA 4 cases, azathioprine (AZA) 3 cases, methotrexate (MTX) 1 case, and thalidomide 1 case. Nine patients received more than one immunosuppressant. Intrathecal injection of dexamethasone 10 mg and methotrexate 10 mg was administrated in 28 patients.

Six severe and/or refractory p-NBD patients, with a poor baseline disability status (mean Rankin score was 4), received biological agents, including infliximab (IFX) in 4 cases, tocilizumab (TCZ) in 1 case, and interferon (IFN)-*α*2a in 1 case (Table 5). After a median follow-up of 21.5 months, all the patients achieved clinical improvements. The Rankin score was significantly decreased to (2.2 ± 0.8) (*P* = 0.002). Additionally, follow-up MRI showed the lesions significantly reduced in two of them. Furthermore, the dosage of corticosteroids was tapered from prednisone ≥ 1 mg/kg/d to 0-10 mg/d, and immunosuppressants were tapered in number and dosage in 3 (50%) and 6 patients (100%), respectively. No serious adverse events were observed.

Overall, with a median follow-up of 28 months (4 to 156 months), 22 out of 36 patients (61.1%) achieved clinical improvements, including the four patients treated with CsA after the diagnosis of p-NBD. 10 patients (27.8%) relapsed and 4 patients died (the mortality rate was 11.1%). Six patients were lost to follow-up.

## 5. Discussion

p-NBD accounted for 67.7% of neuro-Behcet's disease in our cohort, and the proportion and the male predominance (59.5%) was similar to the data reported [11, 12, 17]. The neurological symptoms usually occur after the BD diagnosis is established. However, in 6 patients (14.3%), the neurological manifestations preceded the diagnosis of BD in our series. This emphasizes that rheumatologists and neurologists must be aware of this possible rare cause of neurological symptoms.

As previously reported, the most frequently affected area of p-NBD was the brainstem [4]. Hemicerebrum, spinal cord, and cerebellum can also be involved. Nearly one-third of the patients suffered from multiple lesions. In our study, cervical cord involvement was more common in spinal cord lesions, while previous research has shown that thoracic cord was more susceptible [18]. The difference may be due to different race or study design. The most common initial manifestations of p-NBD were pyramidal signs and headaches, as described in the literature [7]. Psychological and behavioral change showed a high incidence in our study. Atypical symptoms may lead to misdiagnosis.

CSF examination plays an important role in the diagnosis of p-NBD. Our data suggested that most p-NBD patients tend to have normal intracranial pressure and CSF pleocytosis. The pleocytosis pictures of CSF can present as neutrophil-predominant or lymphocyte-predominant depending on the course of the disease [7]. Our data showed a higher CSF-OCB-positive rate in p-NBD than the previous reports [8, 11]. All these CSF-OCB-positive cases in our cohort were in an acute course and with high disease activity. As a previous study showed CSF oligoclonal IgA and IgM may be helpful in monitoring CNS disease activity in NBD [19]. A further study to monitor CSF-OCB in both active and remission periods of p-NBD would be more valuable. Two cases showed high-level proinflammatory cytokines in accordance with active disease. Proinflammatory cytokines such as IL-6 can be markers of disease activity or prognosis [20].

MRI has obvious advantages in displaying small vessel lesions. The most commonly affected site was the midline structure, such as the brainstem. Most lesions in p-NBD were hyperintense on T2-weighted MRI. The previous study indicated that brainstem atrophy, especially in the absence of cortical atrophy, is highly specific to the diagnosis of NBD [21]. Meanwhile, high levels of proinflammatory cytokines, especially IL-6, might be important for the induction of apoptosis of neurons in p-NBD [22]. In our study, one case showed brainstem atrophy on MRI with high-level CSF-IL-6. Given the complexity of the disease, the measurement of a single cytokine does not provide sufficient discrimination to assess the correlation of imaging changes and disease courses [23].

Our data revealed a high frequency of ocular involvement in p-NBD. Ocular involvement, like uveitis, retinitis, and scleritis, is a “warning sign” for predisposition to parenchymal involvement in BD. The recent study from Turkey [9] also showed the significant association between posterior uveitis and p-NBD. The mechanism of this phenomenon has not been clarified. A meta-analysis showed that the risk of developing nervous system involvement was significantly higher among BD patients who used CsA compared with those who did not (RR = 8.26, 95% CI = 4.45-15.32) [24]. In our cohort, six cases complicated with ocular involvement treated with CsA before the onset of p-NBD and the immunosuppressants were adjusted after the neurological attack. However, four cases received high-dose methylprednisolone pulse therapy followed by oral prednisone plus CsA after the diagnosis of p-NBD, achieved remission, and remained stable during the follow-up.

Glucocorticoids and immunosuppressants are the cornerstones for the management of p-NBD [25]. However, no treatment option for p-NBD has been supported by randomized trials. CTX was the most commonly used immunosuppressant in our study, and some cases required combination therapy with multiple immunosuppressants. Systemic administration combined with intrathecal injection with MTX and/or dexamethasone was also effective. For the severe and/or refractory p-NBD cases, biological therapy achieved clinical and imaging improvement in addition to successfully tapering immunosuppressants, indicating a potential steroid- and immunosuppressant-sparing effect. Recently, there is growing evidence revealing the efficacy of the biological agents, such as TNF-*α* inhibitors [26], TCZ [27], and IL-1 inhibitor [28], suggesting alternative therapeutic approaches for refractory cases. The international consensus [15] and 2018 EULAR recommendations [25] for p-NBD also recommended anti-TNF therapy to be considered on severe disease as first-line or refractory patients. We assume that earlier and more widespread use of biologics may improve the outcome. We expect more clinical research to confirm this opinion in the future.

In concordance with previous reports, our study showed a poor prognosis in p-NBD. Four patients died from the disease. Early recognition of severe organ involvements is essential for improving prognosis.

Our study presents the clinical characteristics of the largest cohort of Chinese patients over 16 years. It has several limitations. Firstly, since all patients were enrolled from a national referral center, a potential selection bias toward severe cases could not be excluded. Secondly, it was a retrospective study. Given the limited number of cases, we could not clarify the correlation between CsA and the pathogenesis of NBD. A further multicenter case-control study is warranted to overcome these limitations.

## 6. Conclusions

In summary, the parenchymal neurological involvement is a rare complication of BD with a high morbidity and mortality. It has a male predominance and most commonly involves the brainstem. Ocular involvement is a risk factor for p-NBD. Glucocorticoids and immunosuppressants are the major therapies. Although larger studies are still needed to clarify some issues, as safety and sustained response after discontinuation, biological agents may be a useful alternative choice for p-NBD patients with severe organ damage or refractory to conventional therapy.

## Figures and Tables

**Table 1:** Clinical manifestations of BD.

Clinical manifestations	*N* (%)
Oral ulcers	42 (100%)
Genital ulcers	27 (64.3%)
Skin lesions	19 (45.2%)
Ocular involvement	15 (35.7%)
Arthritis	9 (21.4%)
Vascular involvement	6 (14.3%)
Gastrointestinal lesions	3 (7.1%)

**Table 2:** Sites of neurological involvement in p-NBD.

Neurological lesions	*N* (%)
Brainstem involvement	23 (54.8%)
Isolated brainstem	10 (23.8%)
Brainstem+	13 (31.0%)
Hemicerebrum involvement	22 (52.4%)
Spinal cord involvement	5 (11.9%)
Cervical cord	4 (9.5%)
Thoracic cord	1 (2.4%)
Cerebellum	4 (9.5%)
Localization not possible^∗^	3 (7.1%)
More than two sites	13 (31.0%)

^∗^These 3 patients showed only neurological clinical symptoms and abnormal CSF examinations, and in all these 3 cases, MRI was normal.

**Table 3:** Clinical manifestations of p-NBD.

Neurological symptoms	*N* (%)
Pyramidal sign	21 (50.0%)
Headache	14 (33.3%)
Psychological and behavioral change	14 (33.3%)
Irritating cough	13 (31.0%)
Myasthenia	12 (28.6%)
Dysarthria	11 (26.2%)
Movement disorder	9 (21.4%)
Urinary retention	7 (16.7%)
Visual loss	6 (14.3%)
Cognitive dysfunction	5 (11.9%)
Epilepsy	4 (9.5%)
Conscious disturbance	4 (9.5%)
Ataxia	4 (9.5%)
Hemiplegia	3 (7.1%)

**Table 4:** Clinical comparison of p-NBD and BD without neurological involvement^∗^.

Parameters	p-NBD (*n* = 42)	Controls^¥^ (*n* = 84)	*P* value	OR (95% CI)
Age (years, mean ± SD)	36.3 ± 11.6	35.2 ± 10.9	0.601	1.01 (0.98-1.04)
Gender (F/M)	25/17	50/34	1.000	—
Clinical features of BD				
Oral ulceration	42 (100%)	84 (100%)	1.000	—
Genital ulceration	27 (64.3%)	61 (72.6%)	0.337	0.68 (0.31-1.50)
Skin lesions	19 (45.2%)	40 (47.6%)	0.801	0.91 (0.43-1.91)
Gastrointestinal lesions	3 (7.1%)	17 (20.2%)	0.058	0.30 (0.08-1.10)
Ocular involvement^·^	15 (35.7%)	16 (19.0%)	0.041^||^	2.36 (1.03-5.44)
Vascular involvement	6 (14.3%)	10 (11.9%)	0.705	1.23 (0.42-3.66)
Laboratory tests				
ESR (mm/h, median and range)	15 (0-85)	11 (1-80)	0.072	1.01 (0.99-1.03)
CRP (mg/L, median and range)	3.0 (0.14-124)	3.3 (0.13-104)	0.995	1.00 (0.98-1.02)

OR = odds ratio; CI = confidential interval. ^∗^Values are the number (percentage) unless otherwise indicated. ^¥^Eighty-four age- and gender-matched BD patients without neurological involvement were served as control. ^·^Ocular involvement includes uveitis, retinal vasculitis, and scleritis. ^||^*P* < 0.05.

**(a):** 

Case	Age	M/F	History of treatment	Clinical manifestations of p-NBD		
				Symptoms	Lesion sites	CSF tests
1	24	M	GC^a^ CTX	Pyramidal sign, ataxia, irritating cough	Brainstem, cerebellum, spinal cord	Normal
2	45	M	GC^a^ CTX THD	Pyramidal sign, dysarthria, irritating cough	Brainstem, cerebellum	Pro 0.46 g/L, normal pressure, and cytological test
3	35	M	GC^a^ CTX	Pyramidal sign, epilepsy, psychological and behavioral change, disturbance of urine, myasthenia	Brainstem, hemicerebrum, spinal cord	ICP 200 mmH_2_O, WBC 37∗10^6^/L, Pro 1.19 g/L
4	15	M	GC^b^ MTX	Epilepsy, headache	Not possible	ICP 235 mmH_2_O, normal biochemical and cytological test
5	36	M	GC^b^ MTX AZA	Psychological and behavioral change, disturbance of urine, myasthenia	Brainstem, hemicerebrum, spinal cord	WBC 70∗10^6^/L, Pro 0.61 g/L
6	15	F	GC^a^ CTX CsA	Dysarthria, irritating cough, psychological and behavioral change	Hemicerebrum	ICP 246 mmH_2_O, normal biochemical and cytological test

**(b):** 

Case	Treatment of biological agents				Follow-up		
	Biological agents	Dose	Course	Drug combination	Symptoms	Side effects	Period (months)
1	IFX	3 mg/kg	4 times	GC^c^ CTX	Improved	None	31
2	IFX	5 mg/kg	5 times	GC^c^ CTX	Improved and ESR/CRP became normal	None	12
3	IFX	3 mg/kg	4 times	GC^c^ AZA	Symptoms and follow-up MRI got improved	None	9
4	IFX	3 mg/kg	5 times	MTX	No more seizures	None	72
5	TCZ	8 mg/kg	10 times	GC^c^ AZA	Symptoms and follow-up MRI got improved	None	11
6	IFN-*α*2a	3 Mu	53 months	GC^c^ CsA	Improved and ESR/CRP became normal	None	104

GC: glucocorticoid; CTX: cyclophosphamide; THD: thalidomide; MTX: methotrexate; AZA: azathioprine; CsA: cyclosporine A; Pro: protein; ICP: intracranial pressure; WBC: white blood cell. ^a^Methylprednisolone pulse therapy. ^b^As prednisone ≥ 1 mg/kg/d or the equivalent dosage of other corticosteroids. ^c^As prednisone 5 mg/d-10 mg/d.

## Data Availability

The data used to support the findings of this study are included within the article.
